# Association between Macular Thickness Profiles and Visual Function in Healthy Eyes: The Singapore Epidemiology of Eye Diseases (SEED) Study

**DOI:** 10.1038/s41598-020-63063-y

**Published:** 2020-04-09

**Authors:** Stanley Poh, Yih-Chung Tham, Miao Li Chee, Wei Dai, Shivani Majithia, Zhi Da Soh, Eva K. Fenwick, Yijin Tao, Sahil Thakur, Tyler Hyungtaek Rim, Charumathi Sabanayagam, Ching-Yu Cheng

**Affiliations:** 1 Singapore Eye Research Institute, Singapore National Eye Centre, Singapore, Singapore; 2 Ophthalmology & Visual Sciences Academic Clinical Program (Eye ACP), Duke-NUS Medical School, Singapore, Singapore; 3 Department of Ophthalmology, Yong Loo Lin School of Medicine, National University of Singapore and National University Health System, Singapore, Singapore

**Keywords:** Epidemiology, Medical imaging, Epidemiology

## Abstract

This study aimed to evaluate the association between optical coherence tomography (OCT)-measured retinal layer thickness parameters with clinical and patient-centred visual outcomes in healthy eyes. Participants aged 40 and above were recruited from the Singapore Epidemiology of Eye Diseases Study, a multi-ethnic population-based study. Average macular, ganglion cell-inner plexiform layer (GCIPL), and outer retinal thickness parameters were obtained using the Cirrus High Definition-OCT. Measurements of best-corrected visual acuity (BCVA) and 11-item visual functioning questionnaire (VF-11) were performed. Associations between macular thickness parameters, with BCVA and Rasch-transformed VF-11 scores (in logits) were assessed using multivariable linear regression models with generalized estimating equations, adjusted for relevant confounders. 4,540 subjects (7,744 eyes) with a mean age of 58.8 ± 8.6 years were included. The mean BCVA (LogMAR) was 0.10 ± 0.11 and mean VF-11 score was 5.20 ± 1.29. In multivariable regression analysis, thicker macula (per 20 µm; β = −0.009) and GCIPL (per 20 µm; β = −0.031) were associated with better BCVA (all p ≤ 0.001), while thicker macula (per 20 µm; β = 0.04) and GCIPL (per 20 µm, β = 0.05) were significantly associated with higher VF-11 scores (all p < 0.05). In conclusion, among healthy Asian eyes, thicker macula and GCIPL were associated with better vision and self-reported visual functioning. These findings provide further understanding on the potential influence of macular thickness on visual function.

## Introduction

Optical coherence tomography (OCT) is a non-invasive imaging technique and an essential clinical assessment tool in retinal diseases^1–3^. Using this technique, recent studies have shed insights on variations of macular thickness across gender, age, ethnicity, axial length and refractive error profiles^4–8^.

A series of past studies has shown that changes in the retinal or its sublayer’s thickness in diseased eyes were associated with visual acuity and function^9–14^, and macular thickness has thus been proposed as a potential surrogate for visual function. Previous studies also reported poorer vision in cases who presented with signs of disrupted retinal layers such as the retinal inner layers, external limiting membrane or the ellipsoid zone^15–17^. The association between macular thickness and self-reported visual functioning has only been evaluated in diseased eyes such as age-related macular degeneration^18^. However, the associations between macular thickness with visual acuity and visual functioning have yet been evaluated in healthy eyes. It is postulated that a thicker retina is correlated with better vision in non-pathological eyes, but this has not been evaluated comprehensively.

Hence, the purpose of our study was to evaluate the association between macular thickness and visual acuity and visual functioning, in a multi-ethnic Asian population. The findings from this study may provide further understanding on the relationship between retinal anatomical structure and visual function.

## Methodology

### Study population

The Singapore Epidemiology of Eye Diseases (SEED) Study is a cross-sectional population-based study in Singapore and comprises of adults from three major ethnicities: Chinese, Malay, and Indian. The methodology of the SEED study has been previously described^19–21^. Briefly, participants aged 40–80+ years residing in the Southwestern part of Singapore were recruited and underwent standardised ocular and systemic examinations. Our study population is made up of 3,353 Chinese from the baseline visit in year 2009–2011 (response rate 72.8%), 1,901 Malays from the 6-year follow up visit in 2011–2013 (response rate 72.1%), and 2,200 Indians from the 6-year follow up visit in year 2013–2015 (response rate 75.5%). The baseline visits in the Malay and Indian population did not undergo OCT assessments and hence were not included in this study. This study follows the principles of the Declaration of Helsinki with ethical approval obtained from SingHealth Centralised Institutional Review Board. Written informed consent was obtained from all participants.

### Eligibility criteria

All participants who underwent an OCT scan were included in our study. Exclusion criteria included poor fundus photo quality e.g. dense media opacity and artefacts, low OCT signal strength of <6, history of glaucoma or high cup-disc ratio of >0.75, previous retinal procedures such as laser photocoagulation, retinal surgery, intravitreal injection, and pre-existing retinal diseases such as diabetic retinopathy, age-related macular degeneration, epiretinal membrane and macular hole.

### Ocular and systemic examinations

A standardised examination protocol was used across all three ethnic groups^8^. The presenting visual acuity (VA) was recorded and subjective refraction subsequently performed to ascertain best-corrected VA (BCVA) of each participant. Both parameters were measured in the units of logarithm of the minimum angle of resolution (LogMAR), using number chart (Lighthouse International, New York, USE) at 4 meters. High myopia was defined as spherical equivalent of −5 dioptres or more.

A detailed interviewer-administered questionnaire was used to collect information including demographics, ocular history, medical and surgical history and medication use, height, weight, blood pressure and pulse rate. Body mass index (BMI) between 18.5 to 25 was defined as normal, <18.5 as underweight, 25 to 30 as overweight, and> 30 as obese. A non-fasting venous blood sample was collected for serum lipids, glycosylated haemoglobin A1c (HbA1c), creatinine, and random glucose. Diabetes was defined as random glucose ≥ 11.1 mmol/L, HbA1c ≥ 6.5%, use of diabetic medications and/or self-reported history. Hypertension was defined as systolic blood pressure ≥ 140 mmHg, diastolic blood pressure ≥ 90 mmHg, use of anti-hypertensive medications and/or self-reported history. Hyperlipidaemia was defined as total cholesterol ≥ 6.2 mmol/L, use of lipid-lowering medications and/or self-reported history. Cardiovascular disease (CVD) history was defined as self-reported history of angina, heart attack and/or stroke. Lastly, low socioeconomic status was defined as fulfilling all three criteria of primary education or below, monthly income <2,000 SGD and residing in 1 to 2-room public housing flat.

### Visual functioning assessment

The VF-11 questionnaire was used to assess the impact of retinal thickness parameters on subjects’ vision functioning^22^. The VF-11 is a modified version of VF-14 which has been validated to suit the local Singapore cultural context. The questionnaire was administered by interviewers fluent in English, Chinese, Malay and Tamil. The VF-11 assesses participants’ ability to perform activities of daily living, such as reading the newspaper, reading street signs, recognising friends, seeing stairs, watching television, cooking, and driving during the day or night. Items 1–9 were given a numerical grading from 0 to 4, where 0 represents no difficulty in performing such activity, and 4 represents inability to perform that activity, while items 10 and 11 were rated from 0 to 2. A non-applicable option for each item was available if participants did not do the activity for reasons other than their vision, and these data were excluded from the analyses.

Rasch analysis, a form of Item Response Theory, was applied to assess the psychometric properties of the VF-11, in which raw VF-11 scores are transformed to estimates of interval level Rasch measures, expressed in log of the odds units or logits^23^. The scores were reversed so that higher scores represented better visual functioning. We used the overall VF Rasch-transformed score derived from items 1–9 of VF-11 questionnaire excluding item 10 and item 11 (‘driving during the day’ and ‘driving at night’) as Rasch analysis of the VF-11 revealed these two items to have high level of misfit.

### Spectral-domain optical coherence tomography imaging

**Figure 1:**
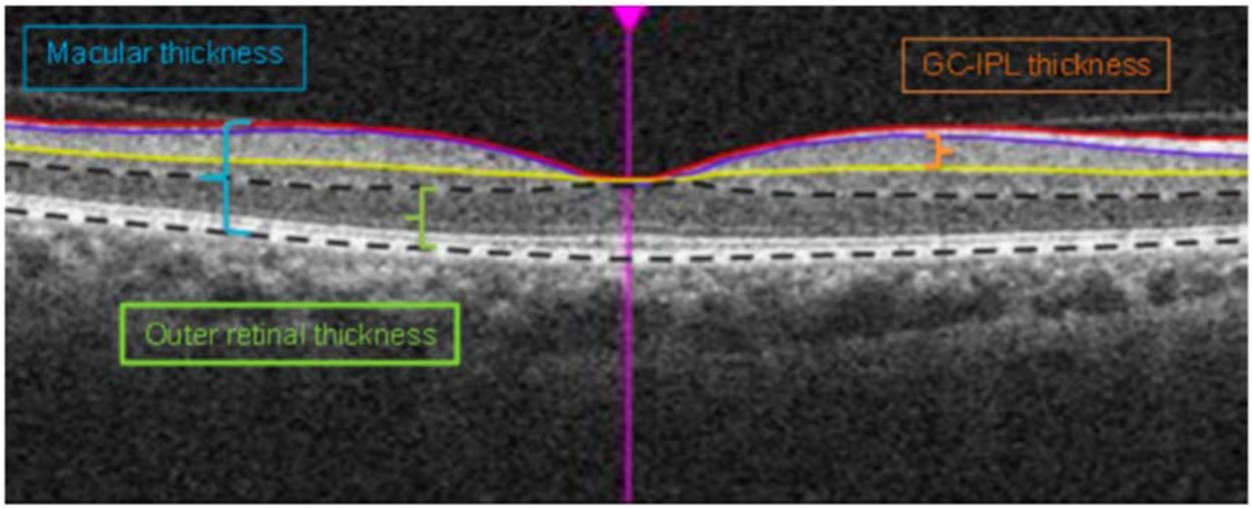
Optical coherence tomography of the macula showing a horizontal B-scan across the fovea. Blue bracket represents macula thickness; Orange bracket represents ganglion cell-inner plexiform layer; Green bracket represents outer retinal thickness.

Cirrus High-Definition OCT (HD-OCT, Carl Zeiss Meditec, Dublin, California, USA) was used to capture images of the macula following pupil dilation. In each study eye, a macular cube scan of 6 × 6 mm^2^ area centred on the fovea was acquired based on the 512 × 128 protocol and automatically analysed by the Cirrus HD-OCT software V6.5. The macula is subdivided into nine macular subfields as defined by the Early Treatment Diabetic Retinopathy Study (ETDRS) with the foveal central subfield defined as central 1 mm diameter, and the inner ring and outer ring with diameters of 3 mm and 6 mm, respectively. Both inner and outer rings are further divided into superior, inferior, nasal and temporal quadrants. The average overall macular thickness was measured from the internal limiting membrane to the retinal pigment epithelium (RPE) of the macular cube. The average ganglion cell-inner plexiform layer (GCIPL) thickness and outer retinal thickness were measured across an elliptical annulus within the 6 × 6 mm^2^ area. GCIPL was segmented from the outer boundaries of retinal nerve fibre layer to the inner plexiform layer, and outer retinal layer was segmented from the outer plexiform layer to the RPE layer (Fig. 1). The four main exposure variables evaluated were foveal central subfield thickness, average macular thickness, average GCIPL thickness, and average outer retinal thickness.

### Statistical analysis

All statistical analyses were performed using Stata 14.0 (StataCorp LP, College Station, TX). Eye-specific analysis data were used. Mean and standard deviation were reported for continuous variables, while frequency and percentage were reported for categorical variables. Univariate linear regression analysis was performed to examine the associations between demographic, systemic and ocular factors with the two outcomes of interest, BCVA and VF-11 Rasch-transformed scores. Variables significantly associated with the two outcomes in univariate analysis (P < 0.05, shown in Supp Tables 1 & 2) and potential confounders (based on prior clinical knowledge, including cataract and axial length) were further included in the multivariable regression models. Multivariable linear regression models with generalized estimating equation (GEE) were used to account for correlation between pairs of eyes.

## Results

**Figure 2:**
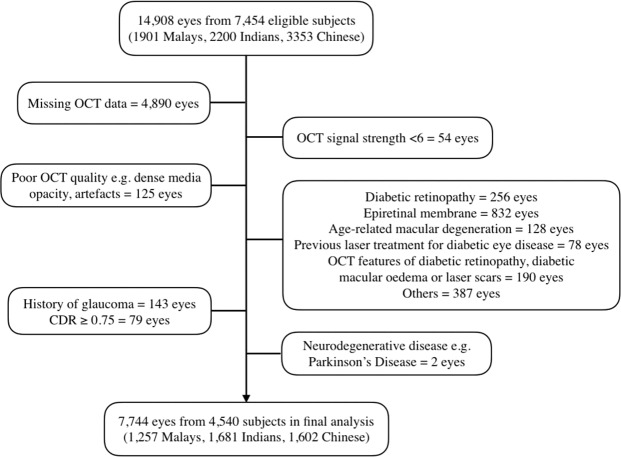
Flowchart showing the inclusion and exclusion processes of this study.

As shown in Fig. 2, after excluding subjects with pre-existing retinal diseases or glaucoma, missing OCT data, poor OCT signal strength or quality and neurodegenerative diseases, a total of 4,450 individuals and 7,744 eyes were included in our analysis. Table 1 shows the population demographics, systemic and ocular characteristics of participants. The mean age of the included sample was 58.6 ± 8.6 years, with 2,333 (51.4%) females. 1,130 (24.9%) had diabetes, 2,700 (59.5%) had hypertension, 2,297 (52.5%) had hyperlipidaemia and 366 (8.1%) had history of CVD. The mean VF-11 score was 5.20 ± 1.29 logits, and the mean BCVA was 0.10 ± 0.11. The mean central subfield thickness was 246.1 ± 21.5 µm, average macular thickness was 276.0 ± 13.4 µm, average GCIPL thickness was 80.4 ± 7.1 µm, and average outer retinal thickness was 123.3 ± 8.5 µm.

**Table 1:** Characteristics of study sample.

Variables	Summary Measure*
**Demographics/systemic characteristics (n = 4,540)**	
Age, years	58.8 (8.6)
Female, n (%)	2,333 (51.4)
Ethnicity, n (%)	
Malay	1,257 (27.7)
Indian	1,681 (37.0)
Chinese	1,602 (35.3)
Low socioeconomic status^†^, n (%)	170 (3.8)
Body mass index, kg/m^2^	
Underweight	155 (3.4)
Normal	2,098 (46.3)
Overweight	1,624 (35.8)
Obese	657 (14.5)
Presence of diabetes, n (%)	1,130 (24.9)
Presence of hypertension, n (%)	2,700 (59.5)
Presence of hyperlipidaemia, n (%)	2,297 (52.5)
Self-reported history of CVD (Yes), n (%)	366 (8.1)
VF-11 scores, logit scale^‡^	5.20 (1.29)
**Ocular Characteristics (n = 7,744 eyes)**	
Best-corrected visual acuity, LogMAR unit	0.10 (0.11)
Axial length, mm	23.6 (1.1)
Spherical equivalent, dioptres	−0.13 (2.07)
Refractive status, n (%)	
Myopic	2,229 (30.4)
Emmetropic	2,165 (29.5)
Hyperopic	2,948 (40.2)
High myopia, n (%)	273 (3.72)
Presence of any cataract, n (%)	3,087 (42.8)
Pseudophakia, n (%)	511 (6.6)
Intra-ocular pressure, mmHg	14.7 (2.9)
VCDR, ratio	0.40 (0.12)
**OCT macular parameters, µm**	
Central subfield thickness	246.1 (21.5)
Average macular thickness	276.0 (13.4)
Average GCIPL thickness	80.4 (7.1)
Average outer retinal thickness	123.3 (8.5)

*Data presented as mean (SD) for continuous variables and n (%) for categorical variables.

†Defined as having primary or lower education, individual monthly income <SGD$2000, and residing in 1–2 room public housing flat.

**‡**Rasch transformed VF-11 scores, with higher scores representing better visual functioning.

CVD = cardiovascular disease; LogMAR= Logarithmic of minimum angle of resolution; VCDR = vertical cup-disc ratio; OCT = optical coherence tomography; GCIPL = ganglion cell-inner plexiform layer.

Table 2 shows the association between OCT macular parameters and BCVA. After adjusting for age, gender, ethnicity, socioeconomic status, body mass index, diabetes, hypertension, hyperlipidaemia, CVD, presence of cataract, axial length and OCT signal strength (significant variables in Supp Table 1), each 20 µm increase in thickness in central subfield macula, overall macula, and GCIPL were significantly associated with better BCVA (all p ≤ 0.001).

**Table 2:** Associations between OCT Macular Parameters and Best-Corrected Visual Acuity.

OCT Macular Parameters	Best-corrected visual acuity			
	Model 1		Model 2	
	β (95% CI)	P-value	β (95% CI)	P-value
Central subfield thickness, per 20 µm	−0.017 (−0.021, −0.012)	<0.001	−0.009 (−0.014, −0.004)	<0.001
Average macular thickness, per 20 µm	−0.032 (−0.037, −0.027)	<0.001	−0.009 (−0.014, −0.003)	0.001
Average GCIPL thickness, per 20 µm	−0.075 (−0.088, −0.062)	<0.001	−0.031 (−0.047, −0.014)	<0.001
Average outer retinal thickness, per 20 µm	0.007 (−0.016, 0.003)	0.187	0.000 (−0.010, 0.010)	0.983

Model 1 – univariate analysis.

Model 2 – multivariable analysis adjusted for age, gender, ethnicity, socioeconomic status, body mass index, diabetes, hypertension, cardiovascular disease, hyperlipidaemia, cataract, axial length and OCT signal strength.

β represents change in BCVA (LogMAR), per 20 µm increase in the respective retinal thickness parameters.

OCT = optical coherence tomography; CI = confidence interval; GCIPL = ganglion cell-inner plexiform layer.

Table 3 shows the association between OCT macular parameters and VF-11 scores. In our multivariable model (Model 2) adjusted for age, gender, ethnicity, socioeconomic status, hypertension, hyperlipidaemia, cardiovascular disease, axial length, cataract, intra-ocular pressure and OCT signal strength (significant variables in Supp Table 2), each 20 µm increase in average macular thickness (β = 0.04) and average GCIPL thickness (β = 0.06) were associated with higher VF-11 scores (all p < 0.05). After adjusting for BCVA in Model 3, average macular thickness (β = 0.04) and average GCIPL thickness (β = 0.05) remained significantly associated with higher VF-11 score (all p < 0.05). The association between central subfield thickness and average outer retinal thickness, and VF-11 scores were not significant in multivariable analysis.

**Table 3:** Associations between OCT Macular Parameters and Visual Functioning Index (VF-11) Scores.

OCT Macular Parameters	VF-11 scores‖							
	Model 1		Model 2		Model 3		Model 4	
	β (95% CI)	Percentage change^‡^ (%)	β (95% CI)	Percentage change^‡^ (%)	β (95% CI)	Percentage change^‡^ (%)	β (95% CI)	Percentage change^‡^ (%)
Central subfield thickness, per 20 µm	0.03 (0.01, 0.05)*	+0.6	0.01 (−0.01, 0.02)	+0.1	0.00 (−0.01, 0.02)	+0.1	0.00 (−0.01, 0.02)	+0.1
Average macular thickness, per 20 µm	0.09 (0.06, 0.13)**	+1.8	0.04 (0.02, 0.07)*	+0.8	0.04 (0.01, 0.06)*	+0.7	0.04 (0.01, 0.06)*	+0.7
Average GCIPL thickness, per 20 µm	0.12 (0.07, 0.18)**	+2.3	0.06 (0.02, 0.09)*	+1.1	0.05 (0.01, 0.08)*	+0.9	0.05 (0.01, 0.08)*	+0.9
Average outer retinal thickness, per 20 µm	0.09 (0.06, 0.13)**	+1.8	0.02 (−0.01, 0.05)	+0.4	0.02 (0.00, 0.05)	+0.4	0.02 (0.00, 0.05)	+0.4

Model 1 – univariate analysis.

Model 2 – multivariate analysis adjusted for age, gender, ethnicity, socioeconomic status, hypertension, cardiovascular disease, hyperlipidaemia, axial length, cataract, intra-ocular pressure and signal strength.

Model 3 – Same as Model 2 with additional adjustment of best-corrected visual acuity.

β represents change in VF-11 scores, per 20 µm increase in the respective retinal thickness parameter.

**‖**Rasch transformed VF-11 scores presented in logit scale.

*Denotes P-value <0.05; ** denotes P-value <0.001.

**‡** β as a percentage of the overall adjusted mean.

OCT, optical coherence tomography; CI, confidence interval; GCIPL, ganglion cell-inner plexiform layer.

## Discussion

In our large, multi-ethnic population-based study, we found that a thicker OCT-measured average macular thickness and GCIPL thickness were both independently associated with better BCVA and visual functioning scores. A thicker central subfield thickness was also significantly associated with better BCVA. These findings support our initial hypothesis that in healthy eyes, a thicker macula is associated with better vision and vision-specific functions. This finding may help to partially explain the subtle variation in vision and visual function among non-pathological eyes that is sometimes observed in clinic.

In addition, we found that poor presenting visual acuity was significantly associated with worse VF-11 scores (per 0.1 LogMAR increase, β = −0.01, p < 0.001). Despite this close relationship, association between the respective macular thickness parameters with VF-11 scores remained significant after adjusting for presenting VA. This further substantiates the observed associations. Furthermore, as age and axial length were major determinants of macular and GCIPL thicknesses, we performed subgroup analyses by age and axial length profiles^4,24^. in view of the close correlation. We observed that the associations with BCVA were consistently observed in both younger (<60 years old) and older age groups (≥60 years old) (Supplementary Table 3a). Significant associations were similarly observed in eyes with axial length of <25 mm, but not in long axial length eyes (Supplementary Table 3b). On the other hand, the associations with VF-11 scores only remained significant for those with shorter axial length of <25 mm (Supplementary Table 4b).

To the best of our knowledge, this is a novel finding in healthy eyes. This contrasts with most other studies which looked at pathological eyes where increased macular thickness was generally associated with poorer visual acuity^10,25–27^. For example, a thicker OCT-measured macular thickness in eyes with vein occlusion or uveitis, as shown in the Standard Care vs. Corticosteroid for Retinal Vein Occlusion (SCORE) study and Multicentre Uveitis Steroid Treatment (MUST) trial, was associated with poorer baseline visual acuity. In comparison, our study evaluated this association in healthy eyes, whereby the differences in macular thickness represent a larger physiological variation among normative population, rather than as a sequela to a macular disease. Our finding of a positive correlation between thicker macula with vision and patient-centred visual function suggests that macular thickness could also be one of the determinants for visual outcomes. Our observed finding may be explained by the notion that thicker macula reflects a more abundant cell number especially of the ganglion cell type which functions to transmit crucial visual information from the retina to the brain^28^.

Our analysis showed that within the inner retinal layer, average GCIPL thickness was also significantly associated with better BCVA and visual functioning scores, but the outer retinal thickness was not. This may be because the majority of the ganglion cells are located in the inner retinal layer of the macular and have greater influence on visual function. Overall, our study supports the existing literature that GCIPL thickness is a determinant of visual outcomes, even in healthy eyes.

We also presented a novel finding in which macular and GCIPL thickness were positively associated with self-reported outcomes in healthy eyes, independent of individual’s vision status. One study looked at the correlation between foveal morphology and visual function in participants with neovascular age-related macular degeneration^18^. They reported an inverted U-shaped correlation between central foveal thickness and visual function, assessed using National Eye Institute Visual Function Questionnaire (VFQ) scores, with best visual function peaking at 220 µm of macular thickness. In contrast, our study evaluated healthy eyes and further took into account a range of potential confounders including individual’s presenting vision status. This shows that, other than clinically measured vision status, macular and GCIPL thickness also potentially contributes to good visual functioning. In healthy eyes, macular thickness can possibly aid in other areas crucial for visual functioning such as depth perception, contrast sensitivity, stereo-acuity, and visual fields, further supporting the role of macular thickness as a factor associated with better visual functioning^18,29^. Overall, despite the statistically significant associations observed between macular parameters and visual functions, it should also be noted that the observed effect estimates were generally small. Hence, the clinical impact of these observed associations remained to be evaluated in future studies.

Strengths of our study include a large, multi-ethnic population. Furthermore, measurements were conducted comprehensively and according to a standardized protocol, allowing us to account for a range of potential confounders. Our study is limited by the lack of other parameters that measure quality of vision (e.g. contrast sensitivity) and information on temporality, which limits inference on causality between retinal layer thickness and visual outcome. Thus, future longitudinal studies in this aspect are still warranted. Our study was also limited by some missing responses on VF-11 questionnaire, which ranged from 0.4 to 36.4% by individual question. However, only three items related to difficulty in cooking, playing games and filling out lottery forms had missing responses of >20%. The higher missing rates in these 3 items were unlikely to have impacted our original findings substantially. Lastly, although the GCIPL thickness was measured using an elliptical annulus area at the macula, we acknowledge that the effect of retinal ganglion cell displacement at the macula was still not fully accounted for in our measurements, and might impact inner retina-related evaluation.

In conclusion, our study demonstrates that in an adult Asian population with healthy eyes, thicker macula and GCIPL were associated with better VA and better visual functioning. Our findings suggest that macular and GCIPL thickness parameters may be useful determinants for visual functions.

## Supplementary information

Supplementary Tables.

## References

[1] Chan A (2006). Normal Macular Thickness Measurements in Healthy Eyes Using Stratus Optical Coherence Tomography. Arch. Ophthalmol..

[2] Hee, M. R. & Lin, C. P. Optical Coherence Tomography of the Human Retina. 8. 10.1001/archopht.1995.01100030081025 7887846

[3] Massin P (2002). Retinal Thickness in Healthy and Diabetic Subjects Measured Using Optical Coherence Tomography Mapping Software. Eur. J. Ophthalmol..

[4] Wong, K. H. *et al*. Racial differences and determinants of macular thickness profiles in multiethnic Asian population: the Singapore Epidemiology of Eye Diseases Study. *Br. J. Ophthalmol*. bjophthalmol-2018-312447, 10.1136/bjophthalmol-2018-312447 (2018). 10.1136/bjophthalmol-2018-312447 30097432

[5] von Hanno T (2017). Macular thickness in healthy eyes of adults (*N* = 4508) and relation to sex, age and refraction: the Tromsø Eye Study (2007-2008). Acta Ophthalmol. (Copenh.).

[6] Wakitani, Y., *et al* Macular thickness measurements in healthy subjects with different axial lengths using optical coherence tomography/; Retina. 2003 Apr;23(2):177–82. 10.1097/00006982-200304000-00007 12707596

[7] Wong ACM, Chan CWN, Hui SP (2005). Relationship of Gender, Body Mass Index and Axial Length with Central Retinal Thickness Using Optical Coherence Tomography. Eye.

[8] Dai, W. *et al*. Macular thickness profile and diabetic retinopathy: the Singapore Epidemiology of Eye Diseases Study. *Br. J. Ophthalmol*. bjophthalmol-2017-310959, 10.1136/bjophthalmol-2017-310959 (2017). 10.1136/bjophthalmol-2017-310959 29175970

[9] Nunes S, Pereira I, Santos A, Bernardes R, Cunha-Vaz J (2010). Central retinal thickness measured with HD-OCT shows a weak correlation with visual acuity in eyes with CSME. Br. J. Ophthalmol..

[10] Diabetic Retinopathy Clinical Research Network (2007). Relationship between Optical Coherence Tomography–Measured Central Retinal Thickness and Visual Acuity in Diabetic Macular Edema. Ophthalmology.

[11] Pelosini L (2011). Optical Coherence Tomography May Be Used to Predict Visual Acuity in Patients with Macular Edema. Investig. Opthalmology Vis. Sci..

[12] Arora S, Sachdeva A, Goel T, Singh K, Aggarwal M (2018). To study the correlation of mean macular thickness using optical coherence tomography with distant and near visual acuity in patients of diabetic maculopathy. Int. J. Res. Med. Sci..

[13] Keane PA, Sadda SR (2011). Predicting visual outcomes for macular disease using optical coherence tomography. Saudi J. Ophthalmol..

[14] Chen, L., Liu, M., Xie, A.-M. & Liu, Y. A study on change of macular retinal thickness and its relationship with vision before and after operation to idiopathic macular epiretinal membranes. 10. PMC4694370 26770470

[15] Sun JK (2014). Disorganization of the Retinal Inner Layers as a Predictor of Visual Acuity in Eyes With Center-Involved Diabetic Macular Edema. JAMA Ophthalmol..

[16] Muftuoglu, I. K., *et al* Integrity of outer retinal layers after resolution of central involved diabetic macular edema: Retina. 2017 Nov;37(11):2015–24. 10.1097/IAE.0000000000001459 PMC5509523 28092342

[17] Wang, J.-W. *et al*. Macular integrity assessment to determine the association between macular microstructure and functional parameters in diabetic macular edema. *Int. J. Ophthalmol*., 10.18240/ijo.2018.07.18 (2018) . 10.18240/ijo.2018.07.18 PMC6048347 30046537

[18] Subhi Y, Henningsen GØ, Larsen CT, Sørensen MS, Sørensen TL (2014). Foveal Morphology Affects Self-Perceived Visual Function and Treatment Response in Neovascular Age-Related Macular Degeneration: A Cohort Study. PLoS ONE.

[19] Lavanya R (2009). Methodology of the Singapore Indian Chinese Cohort (SICC) Eye Study: Quantifying ethnic variations in the epidemiology of eye diseases in Asians. Ophthalmic Epidemiol..

[20] Rosman M (2012). Singapore Malay Eye Study: rationale and methodology of 6-year follow-up study (SiMES-2): Rationale and methodology of SiMES-2. Clin. Experiment. Ophthalmol..

[21] Sabanayagam C (2017). Singapore Indian Eye Study-2: methodology and impact of migration on systemic and eye outcomes: SINDI-2 methodology. Clin. Experiment. Ophthalmol..

[22] Lamoureux EL, Pesudovs K, Thumboo J, Saw S-M, Wong TY (2009). An Evaluation of the Reliability and Validity of the Visual Functioning Questionnaire (VF-11) Using Rasch Analysis in an Asian Population. Investig. Opthalmology Vis. Sci..

[23] Las Hayas C, Bilbao A, Quintana JM, Garcia S, Lafuente I (2011). A Comparison of Standard Scoring versus Rasch Scoring of the Visual Function Index-14 in Patients with Cataracts. Investig. Opthalmology Vis. Sci..

[24] Tham, Y.-C. *et al*. Profiles of Ganglion Cell-Inner Plexiform Layer Thickness in a Multi-ethnic Asian Population: The Singapore Epidemiology of Eye Diseases Study. *Ophthalmology*, 10.1016/j.ophtha.2020.01.055 (2020). 10.1016/j.ophtha.2020.01.055 32197910

[25] Scott IU (2009). SCORE Study Report 1: Baseline Associations between Central Retinal Thickness and Visual Acuity in Patients with Retinal Vein Occlusion. Ophthalmology.

[26] Taylor SRJ (2012). The Impact of Macular Edema on Visual Function in Intermediate, Posterior, and Panuveitis. Ocul. Immunol. Inflamm..

[27] Nussenblatt RB, Kaufman SC, Palestine AG, Davis MD, Ferris FL (1987). Macular Thickening and Visual Acuity. Ophthalmology.

[28] Provis JM, Dubis AM, Maddess T, Carroll J (2013). Adaptation of the central retina for high acuity vision: Cones, the fovea and the avascular zone. Prog. Retin. Eye Res..

[29] Agrawal S, Singh V, Bhasker S, Sharma B (2013). Correlation of visual functions with macular thickness in primary open angle glaucoma. Oman J. Ophthalmol..

