# Supplementary material for: Association between Macular Thickness Profiles and Visual Function in Healthy Eyes: The Singapore Epidemiology of Eye Diseases (SEED) Study
Source: Sci Rep. 2020 Apr 9;10:6142. doi: 10.1038/s41598-020-63063-y (PMC7145798; doi:10.1038/s41598-020-63063-y)
Supplement: Supplementary file 1 — Supplementary Tables. [file 41598_2020_63063_MOESM1_ESM.docx]

**Association between Macular Thickness Profiles and Visual Function in Healthy Eyes: The Singapore Epidemiology of Eye Diseases (SEED) Study**

**AUTHORS**

Stanley Poh, MBBS^1†^

Yih-Chung Tham, PhD^1,2†^

Miao Li Chee^1^

Wei Dai, MPH^1^

Shivani Majithia, OD^1^

Zhi Da Soh, MPH^1^

Eva K. Fenwick, PhD^1,2^

Yijin Tao^1^

Sahil Thakur, MS^1^

Tyler Hyungtaek Rim, MD, MBA^1,2^

Charumathi Sabanayagam, MD, PhD^1,2^

Ching-Yu Cheng, MD, PhD^1,2,3*^

† Contributed equally.

| **Supplementary Table 1. Univariable Association between Systemic and Ocular Factors with Best-Corrected Visual Acuity** | | |
| --- | --- | --- |
| **Factors** | **Best-corrected Visual Acuity** | |
|  | **β (95% CI)** | **P-value** |
| Age, years | 0.006 (0.005, 0.006) | <0.001 |
| Female (vs male) | 0.015 (0.009, 0.021) | <0.001 |
| Ethnicity |  |  |
| Malay | Reference |  |
| Indian | 0.006 (0.005, 0.006) | <0.001 |
| Chinese | 0.015 (0.009, 0.021) | <0.001 |
| Low socioeconomic status | 0.006 (0.005, 0.006) | <0.001 |
| Body mass index, kg/m^2^ |  |  |
| Normal | Reference |  |
| Underweight | 0.001 (-0.016, 0.018) | 0.882 |
| Overweight | 0.012 (0.005, 0.019) | 0.001 |
| Obese | 0.032 (0.022, 0.041) | <0.001 |
| Presence of diabetes | 0.042 (0.034, 0.049) | <0.001 |
| Presence of hypertension | 0.042 (0.036, 0.048) | <0.001 |
| Presence of hyperlipidaemia | 0.027 (0.020, 0.033) | <0.001 |
| Self-reported history of CVD, yes | 0.056 (0.041, 0.071) | <0.001 |
| Axial length, mm | -0.012 (-0.015, -0.009) | <0.001 |
| Spherical equivalent, dioptres† | 0.001 (0.000, 0.003) | 0.110 |
| Refractive status† |  |  |
| Emmetropic | Reference |  |
| Myopic | 0.020 (0.012, 0.027) | <0.001 |
| Hyperopic | 0.030 (0.023, 0.037) | <0.001 |
| Presence of any cataract | 0.073 (0.067, 0.079) | <0.001 |
| Intraocular pressure, mmHg | 0.001 (0.000, 0.002) | 0.054 |
| VCDR | 0.004 (-0.020, 0.028) | 0.719 |
| OCT signal strength | -0.027 (-0.030, -0.024) | <0.001 |
| β represents change in BCVA (LogMAR), per unit increase in the respective exposures | | |
| † assessed in subjects without cataract surgery | | |
| CI, confidence interval; CVD, cardiovascular disease; VCDR, vertical cup-disc ratio; OCT, optical coherence tomography | | |

| **Supplementary Table 2. Univariable Association between Systemic and Ocular Factors with Visual Function Index (VF-11) Scores (logits)** | | | |  |
| --- | --- | --- | --- | --- |
| **Factors** | **VF-11 scores** | | | |
|  | **β (95% CI)** | **P-value** | **Percentage Change (%)**‡ | |
| Age, years | -0.03 (-0.04, -0.03) | <0.001 | +0.6 | |
| Female (vs Male) | -0.18 (-0.26, -0.11) | <0.001 | +3.5 | |
| Ethnicity |  |  |  | |
| Malay | Reference |  |  | |
| Indian | 0.74 (0.64, 0.83) | <0.001 | +13.5 | |
| Chinese | 0.63 (0.53, 0.72) | <0.001 | +11.7 | |
| Low socioeconomic status | -1.29 (-1.49, -1.10) | <0.001 | +32.7 | |
| Body mass index, kg/m^2^ |  |  |  | |
| Normal | Reference |  |  | |
| Underweight | -0.23 (-0.44, -0.02) | 0.032 | +4.6 | |
| Overweight | -0.02 (-0.10, 0.06) | 0.661 | +0.4 | |
| Obese | -0.12 (-0.23, -0.01) | 0.040 | +2.3 | |
| Presence of diabetes | -0.07 (-0.15, 0.02) | 0.140 | +1.3 | |
| Presence of hypertension | -0.27 (-0.35, -0.20) | <0.001 | +5.4 | |
| Presence of hyperlipidaemia | -0.10 (-0.18, -0.03) | 0.007 | +2.0 | |
| Self-reported history of CVD, yes | -0.18 (-0.32, -0.05) | 0.008 | +3.7 | |
| Axial length, mm | 0.06 (0.04, 0.09) | <0.001 | +1.2 | |
| Spherical equivalent, dioptres† | -0.01 (-0.02, 0.00) | 0.034 | +0.2 | |
| Refractive status† |  |  |  | |
| Emmetropic | Reference |  |  | |
| Myopic | 0.01 (-0.01, 0.03) | 0.265 | +0.2 | |
| Hyperopic | -0.01 (-0.03, 0.01) | 0.247 | +0.2 | |
| Presence of any cataract | -0.14 (-0.18, -0.09) | <0.001 | +2.6 | |
| Intraocular pressure, mmHg | 0.01 (0.00, 0.01) | 0.016 | +0.1 | |
| VCDR | 0.04 (-0.07, 0.16) | 0.445 | +0.8 | |
| OCT signal strength | 0.02 (0.01, 0.02) | <0.001 | +0.4 | |
| β represents change in VF-11 scores, per unit increase in the respective exposures | | | |  |
| ‡ β as a percentage of the adjusted mean in the reference group for categorical variables and the overall adjusted mean for continuous variables | | | |  |
| † assessed in subjects without cataract surgery | | | |  |
| CI, confidence interval; CVD, cardiovascular disease; VCDR, vertical cup-disc ratio; OCT, optical coherence tomography | | | |  |

| **Supplementary Table 3a. Sub-analysis of Associations between OCT Macular Parameters and Best-Corrected Visual Acuity by Age** | | | | |
| --- | --- | --- | --- | --- |
| **OCT Macular Parameters** | **Best-corrected visual acuity** | | | |
|  | **Age<60 years** | | **Age≥60 years** | |
|  | **β (95% CI)** | **P-value** | **β (95% CI)** | **P-value** |
| Central subfield thickness,  per 20µm | -0.004 (-0.007, -0.001) | 0.014 | -0.017 (-0.028, -0.006) | 0.002 |
| Average macular thickness,  per 20µm | -0.004 (-0.009, 0.001) | 0.131 | -0.013 (-0.024, -0.002) | 0.020 |
| Average GCIPL thickness,  per 20µm | -0.015 (-0.025, -0.004) | 0.005 | -0.050 (-0.086, -0.013) | 0.007 |
| Average outer retinal thickness, per 20µm | -0.001 (-0.008, 0.007) | 0.835 | 0.005 (-0.020, 0.029) | 0.703 |
| Multivariable analysis adjusted for age, gender, ethnicity, socioeconomic status, body mass index, diabetes, hypertension, cardiovascular disease, hyperlipidaemia, cataract, axial length and OCT signal strength | | | | |

| **Supplementary Table 3b. Sub-analysis of Associations between OCT Macular Parameters and Best-Corrected Visual Acuity by Axial Length** | | | | |
| --- | --- | --- | --- | --- |
| **OCT Macular Parameters** | **Best-corrected visual acuity** | | | |
|  | **AL<25mm (n=6,752)** | | **AL≥25mm (n=860)** | |
|  | **β (95% CI)** | **P-value** | **β (95% CI)** | **P-value** |
| Central subfield thickness,  per 20µm | -0.009 (-0.015, -0.004) | <0.001 | -0.003 (-0.010, 0.003) | 0.313 |
| Average macular thickness,  per 20µm | -0.009 (-0.015, -0.003) | 0.001 | -0.001 (-0.013, 0.011) | 0.896 |
| Average GCIPL thickness,  per 20µm | -0.033 (-0.051, -0.015) | <0.001 | -0.003 (-0.028, 0.021) | 0.783 |
| Average outer retinal thickness, per 20µm | 0.001 (-0.010, 0.012) | 0.862 | -0.002 (-0.017, 0.014) | 0.839 |
| Multivariable analysis adjusted for age, gender, ethnicity, socioeconomic status, body mass index, diabetes, hypertension, cardiovascular disease, hyperlipidaemia, cataract, axial length and OCT signal strength | | | | |

| **Supplementary Table 4a. Sub-analysis of Associations between OCT Macular Parameters and Visual Functioning Index (VF-11) Scores by Age** | | | | |
| --- | --- | --- | --- | --- |
| **OCT Macular Parameters** | **VF-11 scores** | | | |
|  | **Age<60 years** | | **Age≥60 years** | |
|  | **β (95% CI)** | **P-value** | **β (95% CI)** | **P-value** |
| Central subfield thickness,  per 20µm | 0.016 (-0.023, 0.055) | 0.425 | -0.001 (-0.028, 0.026) | 0.944 |
| Average macular thickness,  per 20µm | 0.057 (-0.006, 0.119) | 0.075 | 0.025 (-0.027, 0.077) | 0.345 |
| Average GCIPL thickness,  per 20µm | 0.086 (-0.035, 0.207) | 0.165 | 0.035 (-0.028, 0.098) | 0.275 |
| Average outer retinal thickness,  per 20µm | 0.038 (-0.056, 0.132) | 0.432 | 0.021 (-0.027, 0.070) | 0.385 |
| Multivariable analysis adjusted for age, gender, ethnicity, socioeconomic status, hypertension, cardiovascular disease, hyperlipidaemia, axial length, cataract, intra-ocular pressure, signal strength and presenting visual acuity | | | | |

| **Supplementary Table 4b. Sub-analysis of Associations between OCT Macular Parameters and Visual Functioning Index (VF-11) Scores by Axial Length** | | | | |
| --- | --- | --- | --- | --- |
| **OCT Macular Parameters** | **VF-11 scores** | | | |
|  | **AL<25mm** | | **AL≥25mm** | |
|  | **β (95% CI)** | **P-value** | **β (95% CI)** | **P-value** |
| Central subfield thickness,  per 20µm | 0.005 (-0.011, 0.021) | 0.555 | -0.020 (-0.098, 0.057) | 0.607 |
| Average macular thickness,  per 20µm | 0.042 (0.014, 0.070) | 0.003 | 0.031 (-0.108, 0.170) | 0.664 |
| Average GCIPL thickness,  per 20µm | 0.055 (0.014, 0.097) | 0.009 | -0.009 (-0.239, 0.220) | 0.937 |
| Average outer retinal thickness,  per 20µm | 0.023 (-0.005, 0.052) | 0.108 | 0.055 (-0.087, 0.196) | 0.447 |
| Multivariable analysis adjusted for age, gender, ethnicity, socioeconomic status, hypertension, cardiovascular disease, hyperlipidaemia, axial length, cataract, intra-ocular pressure, signal strength and presenting visual acuity | | | | |
